# Outcomes of Hip Avascular Necrosis Following COVID-19 Infection: A Retrospective Analysis of 212 Hips With Minimum Two-Year Follow-Up

**DOI:** 10.7759/cureus.87230

**Published:** 2025-07-03

**Authors:** Adarsh Annapareddy, Praharsha Mulpur, Tarun Jayakumar, Sandeep Boddeda, Aakarsh Mahajan, Vemaganti Badri Narayana Prasad, A.V. Gurava Reddy

**Affiliations:** 1 Orthopaedics, Sunshine Bone and Joint Institute, KIMS-Sunshine Hospitals, Hyderabad, IND

**Keywords:** avascular necrosis, covid-19, femoral head, hip, steroid induced, total hip arthroplasty

## Abstract

Introduction: Avascular necrosis (AVN) of the femoral head has been observed to occur after COVID-19, especially if steroids were taken. However, the knowledge about its progress in these cases is lacking. We aimed to evaluate the progress of a disease after post-COVID AVN and the effect of various treatments.

Methods: This was a retrospective observational study conducted on 118 patients (212 hips) diagnosed with hip AVN following COVID-19 infection between June 2021 and September 2021. Patients were followed up for a minimum duration of 2 years and evaluated based on demographics, Ficat and Arlet staging, corticosteroid usage, and assessment of treatment modality outcomes. Harris Hip Scores (HHS) were recorded at presentation, 3 months, and 2 years.

Results: The mean age of the cohort was 36.8 years (SD: 10.9), with a majority of males (N=103, 87.3%). The mean interval between the diagnosis of COVID-19 and the onset of hip symptoms was 10.8 months (range: 1-24 months; SD: 5.9) and the mean interval between the onset of hip symptoms and the diagnosis of AVN was 3.65 weeks (range: 1-8 weeks; SD: 1.9), indicating the rapid progress of the disease. The majority of them presented with bilateral AVN (79.7%), with Ficat and Arlet Grade 2 (50.9%) and Grade 3 (41%) AVN, respectively. The mean cumulative steroid dose (prednisolone equivalents) was 439.27 mg (SD: 583.29), with a mean duration of 12.76 days (SD: 12.3). Patients managed conservatively showed an initial improvement in HHS from 77.3 (SD: 11) at presentation to 88.4 (SD: 7.6) at 3 months but declined to 81.8 (SD: 9.7) at 2 years (p<0.001). Core decompression alone resulted in a decline from 73 (SD: 13.5) to 69.5 (SD: 12) at 2 years. In contrast, total hip arthroplasty (THA) led to significant improvement from 64.4 (SD: 12.4) to 92.4 (SD: 3.3) at 2 years (p<0.001).

Conclusion: Post-COVID-19 AVN is an aggressive and rapidly progressing condition affecting young individuals, even with relatively low corticosteroid exposure. Conservative treatments provide only transient benefit, while THA offers substantial and sustained functional improvement in advanced AVN. Early identification and timely surgical intervention, where indicated, are crucial to optimizing patient outcomes in clinical practice.

## Introduction

Long-COVID has been reported to affect around 10% of individuals recovering from the SARS-CoV-2 virus during the pandemic [1]. Long COVID refers to multisystemic complications that occur as a sequela after active COVID-19 infections, usually presenting as multisystem involvement. Several case reports and small case series have reported an increase in avascular necrosis (AVN) of the hip in young adults after COVID-19 infection. Agarwala et al. classified post-COVID AVN as a potential musculoskeletal manifestation of long COVID [1].

The pathophysiology of long COVID involves persistent endothelial dysfunction, microvascular thrombosis, and chronic inflammation, which may predispose to ischemic conditions such as AVN. COVID-19 is primarily a respiratory illness but triggers a systemic inflammatory response with widespread vasculitis, hypercoagulability, and microvascular injury [2-4]. The femoral head has a precarious blood supply, and microvascular insults can result in reduced blood supply and AVN [5]. Furthermore, the incidence of femoral head AVN in India is reportedly higher than in Western populations, indicating potential geographic and demographic susceptibilities [6].

Corticosteroids (oral and intravenous), widely employed during COVID-19 management to counteract cytokine storms, play an additional role in the pathogenesis of AVN. They impair bone microcirculation through lipid metabolism dysregulation, fat embolism, and endothelial damage. The combination of COVID-19-induced endothelial dysfunction and steroid-related vascular compromise may synergistically accelerate AVN development. Shetty et al. have highlighted this concerning interaction between corticosteroid use and COVID-19 in precipitating hip AVN [7]. Agarwala et al. reported a lower dose threshold to develop AVN in patients with a history of COVID compared to non-COVID AVN patients [1].

Although recent studies, including those by Dhanasekararaja et al., have described the clinical course of post-COVID AVN, comprehensive data on its natural history, cumulative steroid exposure thresholds, and long-term treatment outcomes remain limited [8]. It is also important to understand the interval between steroid use and the onset of AVN symptoms. Due to the paucity of reports and clinical data, the real clinical burden of post-COVID AVN is still not clear.

The primary aim of this study was to evaluate the two-year functional outcomes of different treatment modalities such as oral bisphosphonates (BPs), core decompression (CD), and total hip arthroplasty (THA) for post-COVID AVN. The secondary objectives include an assessment of the cumulative steroid dose in post-COVID AVN and the time interval between the diagnosis of COVID and the onset of hip symptoms. We also evaluated the progression of AVN after conservative management and CD.

## Materials and methods

This was a retrospective observational study of patients diagnosed with AVN of the femoral head (unilateral or bilateral) secondary to COVID-19 infection, diagnosed between June 2021 and September 2021, who completed a minimum follow-up of 2 years after diagnosis. This study was approved by the institutional ethical committee (SIEC/2022/479), and informed consent was obtained from all the patients for their data to be used for publication. A total of 130 patients (232 hips) were initially screened. After applying the selection criteria, 118 patients (212 hips) were included in the final analysis. The remaining 12 patients were excluded due to either incomplete follow-up data, prior history of hip pathology, pre-existing steroid use for unrelated conditions, incomplete records, or lost to follow-up.

Inclusion and exclusion criteria

Only patients who completed a minimum follow-up of two years, following the diagnosis and treatment of AVN, were included in this study. All these patients had tested positive for COVID-19 via reverse transcription polymerase chain reaction, and none had prior hip pain or a history of steroid intake for any cause, which could have confounded the development of AVN. We excluded patients who had prior hip pain before COVID-19 treatment, previous surgery in the affected hip, chronic steroid use, active infection in the hip, bladder, chest, skin, or any other area, hemophilia or other bleeding disorders, and progressive neurological disease.

Data collection

We collected demographic data on the patient's age, gender, duration between COVID-19 infection, initial hip symptoms, and duration of hip pain. Patients were categorized based on the Ficat and Arlet staging for AVN of the femoral head [9]. Corticosteroids were administered as supportive treatment for COVID-19 in most patients, either intravenously or orally, depending on the severity and prescription. Steroid type, dose, and mode of administration were also documented. Methylprednisolone was the most commonly used steroid, with other steroids such as dexamethasone, prednisolone, and deflazacort also administered. The net cumulative steroid dose was calculated in terms of prednisolone equivalents.

Diagnosis

Patients presenting with acute hip pain were assessed through plain radiographs, and further evaluation was conducted by MRI as needed. Some patients were already diagnosed with AVN after COVID-19 and visited us for a second opinion and further management. AVN was diagnosed using both plain radiographs and MRIs. The MRI scan included fat-suppressed (FS)-T2, pre- and post-gadolinium T1-weighted imaging. The diagnosis of AVN of the femoral head was established through characteristic findings on MRI. On T1-weighted fast spin-echo (FSE) sequences, AVN manifests as a well-demarcated geographic lesion exhibiting low signal intensity, indicative of bone marrow edema. This area of edema is typically delineated by a distinct hyperintense curvilinear border. On T2-weighted FSE sequences, a highly specific diagnostic feature known as the "double line sign," which comprises two concentric curvilinear lines at the interface between the necrotic and viable bone, is seen.

Treatment

The guidelines for managing AVN of the femoral head based on disease stage are outlined as follows [10]: a) Grade I: Administration of oral BPs, specifically alendronate 70 mg once weekly for 12 months. b) Grade II: Conservative intervention involving BPs and CD with or without bone marrow aspirate concentrate (BMAC). c) Grades III and IV: THA. However, it is imperative to note that CD and BMAC implantation were not performed as per our institutional protocol. All patients undergoing these procedures during the study were treated elsewhere, and our involvement was limited to assessing their two-year follow-up.

Statistical analysis

Normality of data distribution was assessed using the Shapiro-Wilk test. Descriptive data is represented using mean, standard deviation, and range. Categorical data are reported as percentages or proportions. Continuous variables were analyzed using the independent samples t-test and one-way repeated measures ANOVA. Categorical data variables were analyzed using the chi-square test. The p-value of <0.05 was considered significant. Statistical analyses were completed using Statistical Package for Social Sciences (SPSS) version 22 (IBM Corp., Armonk, NY).

## Results

Patient demographics

Of the 130 patients diagnosed with post-COVID AVN, a total of 118 patients, who fulfilled our inclusion and exclusion criteria, were included in this study. We studied 212 hips with AVN in these patients. Table 1 summarizes the demographics and patient presentation information. The average age of the cohort was 36.8 years (range: 21-82 years; SD: 10.9), with males outnumbering females (n=103, 87.3%). The mean interval between the diagnosis of COVID-19 and the onset of hip symptoms was 10.8 months (range: 1-24 months; SD: 5.9), and the mean interval between the onset of hip symptoms and the diagnosis of AVN was 3.65 weeks (range: 1-8 weeks; SD: 1.9), indicating the rapid progress of the disease.

**Table 1:** Summary of the demographics of the study population NA, not applicable

Demographics	Value	Range
Mean age in years (SD)	36.84 (10.98)	21-82
Gender		
Female (numbers, %)	15 (12.7%)	NA
Male (numbers, %)	103 (87.3%)	NA
Side involvement		
Unilateral (numbers, %)	24 (20.3%)	NA
Bilateral (numbers, %)	94 (79.7%)	NA
Mean interval between COVID diagnosis and the onset of symptoms (in months)	10.8 (5.94)	1-24
Mean interval between onset of symptoms and diagnosis of AVN (in weeks)	3.65 (1.93)	1-8
Mean Harris Hip Score at presentation	73.72 (12.63)	19-96
Mean Harris Hip Score at final follow-up	83.75 (10.3)	42-96

MRI findings

The majority of the post-COVID patients presented with bilateral AVN of the femoral head at the time of presentation (n=94, 79.7%). The side distribution based on MRI findings is presented in Table 2. Most patients presented with a Grade 2 (n=108, 50.9%) or Grade 3 (n=87, 41%) Ficat and Arlet stage of AVN. Nine (7.6%) patients had Grade 4 AVN. None of our patients exhibited any symptoms related to other joints such as the shoulder, knee, or ankle that suggested AVN.

**Table 2:** Details of the steroids used for the treatment of COVID-19 in our patient population

	YES	NO
Oral steroids	116 (98.3%)	2 (1.7%)
Intravenous steroids	39 (33.1%)	79 (66.9%)
	MEAN	SD
Mean duration of steroid use (in days)	12.76	12.32
Mean cumulative dose of steroid (in mg) (prednisolone equivalents)	439.27	583.29

Steroid intake

**Figure 1:**
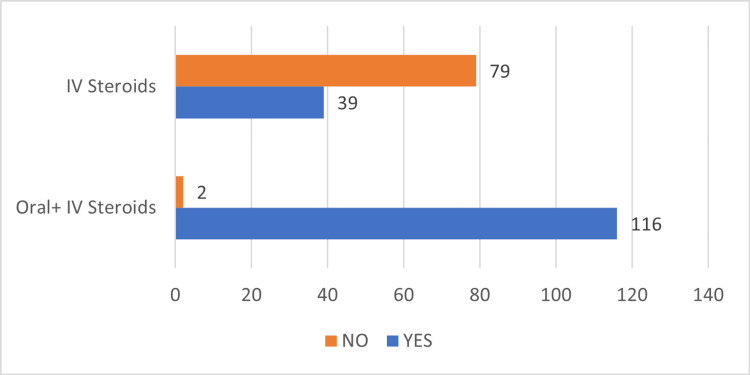
Trends in steroid usage pattern in our cohort of COVID-19 patients

The mean cumulative dose of steroid (prednisolone equivalents) intake in our cohorts was 439.27 mg (range: 32-2680 mg), with a mean duration of 12.76 days (SD: 12.3). The majority of the patients were on IV steroids initially, followed by a gradually tapering course of oral steroids (Table 2, Figure 1).

Treatment modalities

The majority of patients (n=79, 67%) were treated conservatively with oral BP, physiotherapy, and guarded weight bearing. The patients who underwent surgery included nine (7.6%) patients with CD (among whom three patients had additional BMAC implantation) and 30 (25.4%) patients who underwent THA. The outcomes of all these patients were assessed over a two-year period using the Harris Hip Score (HHS) (Table 3).

**Table 3:** Trend of Harris Hip Score at different follow-up times after various treatment modalities *One-way repeated measures ANOVA

	At presentation	3 months	2 years	p-value
Conservative	77.3 (11)	88.4 (7.6)	81.8 (9.7)	<0.0001*
Core decompression	73 (13.5)	81.8 (7.5)	69.5(12)	0.127*
Core decompression and bone marrow aspirate concentrate	73.3 (9.2)	82 (7.2)	85.3 (9.4)	NA
Total hip arthroplasty	64.4 (12.4)	60.8 (13.1)	92.4 (3.3)	<0.0001*
Overall	73.7 (12.6)	81 (15)	83.7 (10.2)	<0.0001*

Clinical outcomes of various treatment modalities

**Figure 2:**
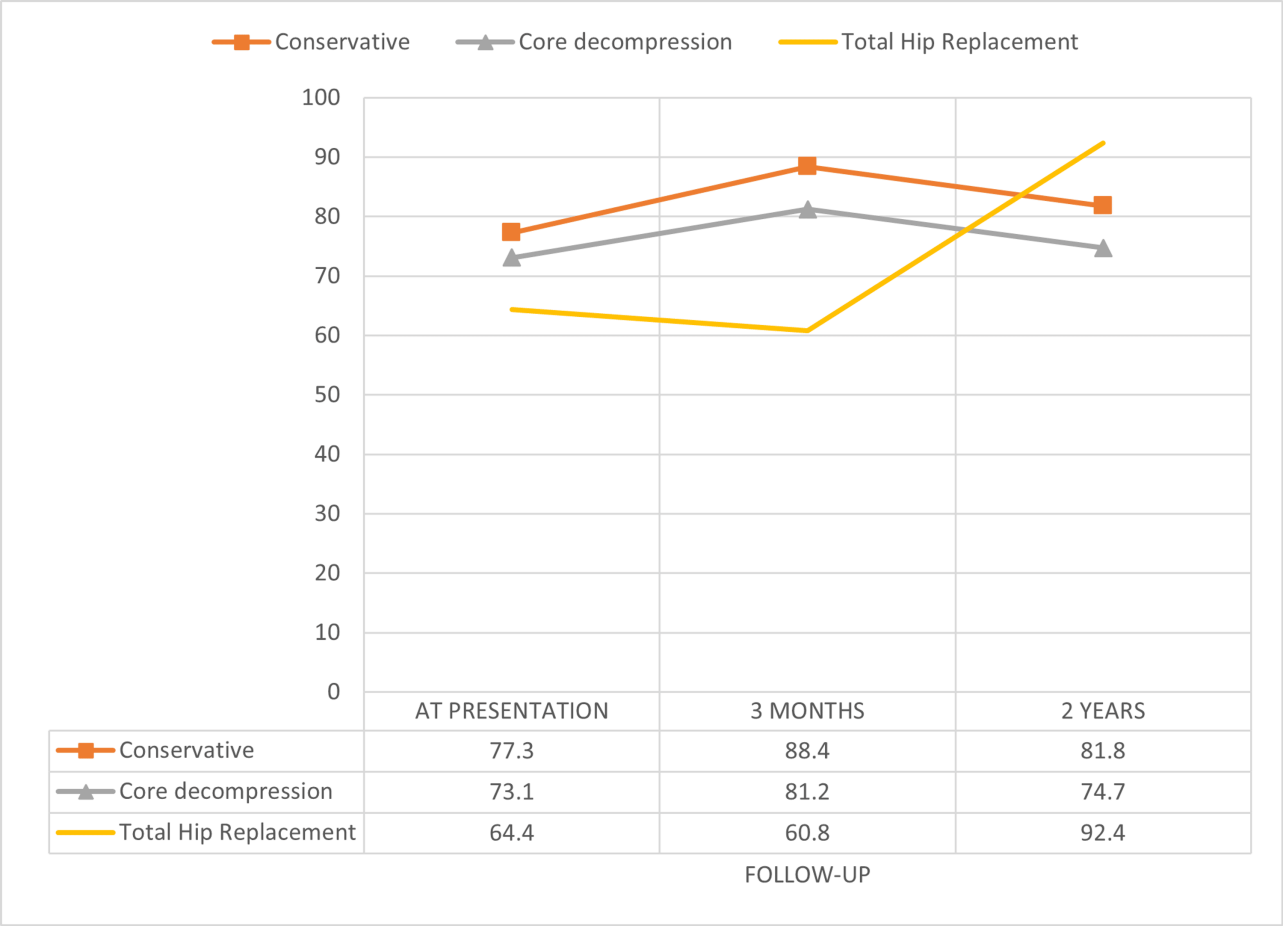
Functional outcome assessed using Harris Hip Score at presentation and subsequent follow-ups with different modalities

Patients who received conservative management initially had an average HHS of 77.3 (SD: 11) at presentation, which showed improvement at the three-month follow-up, with an HHS of 88.4 (SD: 7.6). However, this improvement was temporary, as their function gradually declined over time, resulting in a two-year follow-up HHS of 81.8 (SD: 9.7), which was highly significant (p<0.001). A similar pattern was observed in patients who underwent CD, as their HHS scores at presentation were 73.0 (SD: 13.5), which dropped down to 69.5 (SD: 12) at two-year follow-up. At the same time, patients who underwent CD with BMAC implantation showed improvement in pain scores at subsequent follow-up up to two years. Patients who underwent THA had the lowest initial HHS scores at presentation, with an average of 64.4 (SD-12.4). At the three-month follow-up, their scores decreased further to 60.8 (SD: 13.1). However, they experienced substantial improvement at the two-year follow-up, with an HHS of 92.4 (SD: 3.3), which was highly significant (p<0.001) (Table 3, Figure 2).

## Discussion

The emergence of AVN of the hip in patients who had COVID-19 is a serious concern that warrants further investigation. We observed a notable association between COVID-19 and an increased risk of AVN of the hip. This finding aligns with recent research that highlights COVID-19's pro-inflammatory nature, leading to cytokine storms and microvascular dysfunction, which can compromise blood flow to the femoral head and contribute to the development of AVN [1,2]. Table 4 summarizes the existing literature on the topic of post-COVID AVN of the hip. The current published literature is mostly based on case reports and small case series, with a limited follow-up. Hence, our study provides crucial information and may act as a reference for future researchers [11-22].

**Table 4:** Summary of the published reports on COVID-19 avascular necrosis of the femoral head NA, not applicable; NR, not reported; THA, total hip arthroplasty

S. no	Author	Year	Type of study	No. of patients	Follow up	Age (years)	Gender	Cumulative steroid dose (prednisolone equivalents)	Duration of steroid	Management
					(in months)	Mean ± SD	M/F	Mean (in mg)	Mean (in days)	
1	Agarwala et al. [11]	2022	Case series	48	10	NR	NR	841.3	NA	Conservative
2	Agarwala et al. [1]	2021	Case report	3	1-3	37.3±1.3	3/0	758	22.5	Conservative
3	Annam et al. [12]	2022	Case report	2	NR	48±21	2/0	1470	19	2: Core decompression, 2: THA
4	Ardakani et al. [13]	2022	Case report	5	NR	38.4±14.5	2/3	1695.2	16	THA
5	Dhanasekararaja et al. [8]	2022	Case series	22	NR	38.8	20/2	811	19.6	Conservative, 3: THA
6	Etta et al. [14]	2022	Case report	1	NR	38	1/0	NA	NA	Core decompression
7	Jain et al. [15]	2022	Case report	1	6-13	42	1/0	NA	22	THA
8	Kamani et al. [16]	2022	Case report	1	NR	40	1/0	NA	NA	Core decompression
9	Kandari et al. [17]	2022	Case series	11	7	45.8±8.3	9/2	1855.6	22	Conservative, 1: Core decompression, 2: THA
10	Kingma et al. [18]	2022	Case report	1	NR	60	1/0	NA	NA	THA
11	Maharjan et al. [19]	2022	Case report	1	2	22	0/1	NA	NA	Core decompression
12	Mahran et al. [20]	2022	Case report	4	NR	27.8±6.4	2/2	NA	NA	Core decompression, 1: THA
13	Sulewski et al. [21]	2021	Case series	3	5-10	66.7±3.4	1/2	NA	NA	1: Conservative, 2: THA
14	Yilmam et al. [22]	2021	Case report	1	NR	44	0/1	NA	45	Conservative (hyperbaric oxygen)

Among the 118 patients (with 212 hips and post-COVID AVN) included in the study, the majority of subjects were young (mean age of 36.8 years) and male (87.3%). The substantial number of hips (36 hips, 16.98%) in 30 cases required THA due to end-stage AVN at the time of presentation to us. CD of the femoral head was performed on 11 hips (in eight cases), while one hip received BMAC therapy along with CD in hips with pre-collapsed stage (Stages 1 and 2) to relieve pain and restore hip function and to alleviate or postpone the need for THA. These findings are consistent with previous studies that have advocated surgical approaches for later-stage AVN cases [3,4]. Hip preservation techniques (CD ± BMAC therapy [23]) and muscle pedicle grafting [24] are indicated for pre-collapse stages of AVN. Our results demonstrate that significant improvement in the HSS was observed a) in the pre-collapse stage (Stages 1 and 2) with conservative treatment and b) in the post-collapse stage (Stages 3 and 4) with THA. The results of CD alone did not show a significant improvement in the HSS, possibly because additional cellular therapy such as BMAC was not used in the majority of our cases. BMAC in addition to CD, being effective in the pre-collapsed stage of AVN, should be considered, especially in younger patients [23].

The average duration between the diagnosis of COVID-19 and the onset of AVN symptoms, in our study, was approximately 10.8 months. This delayed manifestation suggests a possible latent period during which the disease process is initiated but takes time to become clinically apparent. The prolonged interval between COVID-19 infection and the onset of AVN raises questions about the potential underlying mechanisms involved in the pathogenesis of the disease. However, the interval between the onset of symptoms and the diagnosis of AVN is much shorter, which is about 3.65 weeks in our study. This may be due to clinicians' increased awareness of this condition and the easier availability of imaging facilities such as MRI. Bilateral involvement of the hip is a norm in the majority of these cases, as 79.7% of our cohort had bilateral disease on MRI/X-rays.

A larger number of our cases (92.4%) had mild to moderate degrees of AVN (Grade II/III) and thus were offered either conservative treatment or hip preservation surgery (CD ± BMAC). In contrast, the remaining patients with advanced stages of hip arthritis were treated with THA. All the cohorts in our study received BP and other supportive medications as initial management, as BP is known to inhibit bone resorption and has been used to manage AVN, aiming to slow down the progression of bone damage and alleviate symptoms [11]. Encouragingly, 90% of patients showed improvement within three months of initiating BP therapy in the early or pre-collapsed stage of AVN. However, we found that the improvement with BP treatment is short-lived, as functional scores declined during the two-year follow-up period, a finding similar to that of the recent systematic review by George et al. [10].

We also studied the potential role of corticosteroids in the development of AVN after COVID-19 infection. Dayan et al. [25] and Birla et al. [5] reported a prevalence of 3% to 38% of steroid-induced AVN, with this entity being the second most common cause of hip AVN after trauma. Most case reports of steroid-induced AVN involve high-dose pulsed steroids, with dexamethasone and methylprednisolone being the most commonly used drugs. Furthermore, McAvoy et al. studied the dose-event relationship in patients receiving corticosteroids after stem cell transplants. They reported that an exposure of <3870 mg of prednisone was associated with a four times higher risk of AVN [26]. However, in our cohorts, the mean cumulative dose of corticosteroids was lower, with an average of 439.27 mg and a mean exposure time of less than two weeks. This may suggest that even short-term corticosteroid use in COVID-19 treatment may contribute to AVN development, and the addition of COVID-19 may be a contributing factor. Dhanasekararaja et al. also reported rapid progression of AVN after COVID-19 with a lower cumulative dose of corticosteroids (mean of 811 mg (range: 200-2100 mg)), which is similar to the findings in our study [8].

We observed a unique disease progression in patients with COVID-19-related AVN, where patients in stage II or III exhibited low functional scores, increased soft-tissue edema on MRI, and high pain scores. This observation contrasts with typical idiopathic AVN, where patients usually progress to THA at later stages of the disease, with evident articular destruction and severe femoral head malformation on plain radiographs. The unique progression observed in COVID-19-related AVN warrants further investigation and might have implications for treatment decisions [27-29]. We suggest conducting larger multicenter studies and establishing data registries on COVID-19-related AVN to better understand the true global incidence of this disease and to formulate appropriate guidelines for its management. Continuous monitoring and research in this area are essential to improve patient outcomes and tailor treatment strategies to address this emerging clinical challenge.

Strengths and limitations of the study

This is the largest case series reporting AVN following COVID-19, with several newer disclosures. The minimum follow-up duration was longer than almost all existing reports, with a complete assessment of functional status at a minimum of 24 months. The study had a low attrition rate in the assessment of patient-reported outcome measures.

However, we acknowledge certain limitations of the study. First, the study was a retrospective analysis of data. Second, the cohort of patients subjected to CD and BMAC implantation is comparatively small, and all procedures were executed at external institutions in all instances. Consequently, we find ourselves unable to definitively ascertain the advantages or disadvantages associated with CD with or without BMAC. This limitation in sample size may impede our ability to draw conclusive findings. Third, the severity of COVID-19 infection (mild, moderate, and severe) and the length of ICU stay were not evaluated. Finally, radiological progression of the disease was not available in all patients included in the final analysis, as several patients were unwilling to undergo MRI on follow-up or had moved away, making radiological evaluation difficult.

## Conclusions

Our study demonstrates that AVN following COVID-19 disproportionately affects younger patients and often presents with bilateral involvement. Even at relatively low cumulative steroid doses, the risk of AVN remains significant. Conservative treatments offer transient relief in pre-collapse stages, whereas THA remains the most reliable option for post-collapse AVN. These findings advocate for early diagnosis and timely surgical intervention to mitigate disability. Furthermore, the lack of standardized management pathways for post-COVID AVN highlights an urgent need for consensus-driven clinical guidelines and further high-quality research.
